# Investigation and Functional Enrichment Analysis of the Human Host Interaction Network with Common Gram-Negative Respiratory Pathogens Predicts Possible Association with Lung Adenocarcinoma

**DOI:** 10.3390/pathophysiology28010003

**Published:** 2021-01-02

**Authors:** Lydia-Eirini Giannakou, Athanasios-Stefanos Giannopoulos, Chrissi Hatzoglou, Konstantinos I. Gourgoulianis, Erasmia Rouka, Sotirios G. Zarogiannis

**Affiliations:** 1 Department of Physiology, Faculty of Medicine, School of Health Sciences, University of Thessaly, BIOPOLIS, 41500 Larissa, Greece; lgiannakou@med.uth.gr (L.-E.G.); atgiannopoulos@med.uth.gr (A.-S.G.); chatz@med.uth.gr (C.H.); szarog@med.uth.gr (S.G.Z.); 2 Department of Respiratory Medicine, Faculty of Medicine, School of Health Sciences, University of Thessaly, BIOPOLIS, 41500 Larissa, Greece; kgourg@med.uth.gr

**Keywords:** bioinformatics, gram negative bacteria, in silico, intercellular junctions, lung adenocarcinoma, host–pathogen interactions

## Abstract

*Haemophilus influenzae* (*Hi*), *Moraxella catarrhalis* (*MorCa*) and *Pseudomonas aeruginosa* (*Psa*) are three of the most common gram-negative bacteria responsible for human respiratory diseases. In this study, we aimed to identify, using the functional enrichment analysis (FEA), the human gene interaction network with the aforementioned bacteria in order to elucidate the full spectrum of induced pathogenicity. The Human Pathogen Interaction Database (HPIDB 3.0) was used to identify the human proteins that interact with the three pathogens. FEA was performed via the ToppFun tool of the ToppGene Suite and the GeneCodis database so as to identify enriched gene ontologies (GO) of biological processes (BP), cellular components (CC) and diseases. In total, 11 human proteins were found to interact with the bacterial pathogens. FEA of BP GOs revealed associations with mitochondrial membrane permeability relative to apoptotic pathways. FEA of CC GOs revealed associations with focal adhesion, cell junctions and exosomes. The most significantly enriched annotations in diseases and pathways were lung adenocarcinoma and cell cycle, respectively. Our results suggest that the *Hi*, *MorCa* and *Psa* pathogens could be related to the pathogenesis and/or progression of lung adenocarcinoma via the targeting of the epithelial cellular junctions and the subsequent deregulation of the cell adhesion and apoptotic pathways. These hypotheses should be experimentally validated.

## 1. Introduction

*Haemophilus influenza* (*Hi*), *Moraxella catarrhalis* (*MorCa*) and *Pseudomonas aeruginosa* (*Psa*) are common Gram-negative pathogens that affect the human respiratory tract. They have been associated with acute otitis media, sinusitis and bronchitis, exacerbations in COPD patients, bronchiectasis in cystic and non-cystic fibrosis patients, community-acquired pneumonia (CAP) and hospital-acquired pneumonia (HAP) [1,2,3,4].

*Hi* is responsible for CAP and exacerbations of COPD. The non-type b strains are linked to more invasive disease in CAP patients while non-typable strains (NTHi) are mainly responsible for COPD exacerbations. Following the introduction of the vaccine against *Hi* strain b (*Hib*), the incidence of infection due to other strains, such as type f (*Hif*) and NTHi, has increased [5]. More specifically, a study conducted in Europe from 2007 to 2014 reported a 3.3% annual increase in *Hi* invasive disease, with NTHi being responsible for 78% of all cases [6]. Moreover, a recent study in the US indicated a 16% increase during 2009–2015 compared with 2002–2008 [7].

*MorCa* is an opportunistic pathogen of the respiratory tract, commonly associated with otitis media in children and COPD exacerbations in adults [8]. It has been estimated that 10% of all exacerbations of COPD and 2–4 million exacerbations per year in the USA are attributed to *MorCa* [9]. Additionally, cases of *MorCa* and *Hi* co-infection have been reported, where it has been suggested that the two pathogens act synergistically, facilitating each other’s pathogenicity and survival in the respiratory tract [10].

*Psa* is not a common cause of CAP, yet it inflicts more severe disease. It is mostly responsible for HAP, intensive care unit (ICU) acquired pneumonia and opportunistic infections. Further, it is one of the most common multi-drug resistant pathogens isolated in infectious respiratory specimens [4,11]. *Psa* has been associated with more severe clinical manifestations and higher mortality rates in patients with bronchiectasis [12].

Molecular pathways that mediate pathogenicity of gram-negative bacteria, especially *Hi*, *MorCa* and *Psa*, have been the topic of various studies. The *Hi* Lipoprotein H (lph) and Protein E have been shown to facilitate pathogen resistance to host immune response by inhibiting complement factor H and membrane attack complex (MAC), respectively [13,14]. The adhesive protein UspA1 of *MorCa* has been characterized as being important for the adhesion and internalization of pathogens in the epithelium [15]. Lastly, the *Psa* Exoenzyme S (ExoS) has been reported to bind to the Rho GTPases, thus contributing to serum resistance by blocking phagocytosis [16]. Amid all of these findings, there is still a lack of data regarding the broad spectrum of the host–pathogen gene interaction network (interactome) and the functional annotations associated with this network. Moreover, novel findings on pathogen synergy [10] and pathogen resistance to host defense mechanisms [17] further highlight the importance of a detailed analysis of the human-pathogen interactome. We conducted an in silico investigation using bioinformatics tools. Additionally, we identified the enriched gene ontologies (GOs) of the functional annotations associated with the host’s biological processes (BP), cellular components (CC) and associated diseases.

## 2. Materials and Methods

The Host Pathogen Interaction Database version 3.0 (HPIDB 3.0) was used to identify the human proteins interacting with the three pathogens of interest. HPIDB is a database that facilitates the host pathogen interaction (HPI) prediction and analysis, collecting published, experimental molecular HPI data from 12 different databases [18]. The database allows searching based on protein sequence, keywords or homologous HPI. In this study the search was performed using the Keyword tool that further offers four query options. We used the “Taxon name/Species” option and *Haemophilus influenzae, Moraxella catarrhalis* and *Pseudomonas aeruginosa* were inserted as keywords. Non-human proteins interacting with the three pathogens were also identified. Subsequently, out of those, only human proteins were further analyzed.

The identified HPIDB protein names were inserted in the Uniprot database in order to retrieve the names of the corresponding genes [19]. These gene names were then inserted in the ToppFun tool of the ToppGene database so as to perform Functional Enrichment Analysis (FEA) relative to BP, CC and diseases. ToppFun performs FEA of input gene list based on transcriptome, proteome, regulome, ontologies, phenotype, pharmacome and bibliome assuming the whole genome as background [20]. The *p* values were corrected for multiple testing with the Bonferroni and false discovery rate (FDR) methods of Benjamini–Hochberg and Benjamini–Yekutieli to determine statistical significance.

In order to corroborate our findings, we performed the same analysis by inquiring the GeneCodis database. GeneCodis is a bioinformatics platform designed for FEA that integrates functional, regulatory or structural information, searches for frequent patterns in the space of annotations and estimates their statistical relevance [21]. The analysis was performed with respect to BP GOs, CC GOs and KEGG pathways. The hypergeometric *p* values retrieved from the analysis were adjusted using the FDR method of Benjamini–Hochberg. The enrichment significance cut off level for the adjusted *p* value was 0.05 in both databases. All analyses were conducted in February 2020.

## 3. Results

### 3.1. Identification of the Host–Pathogen Interactomes

**Figure 1:**
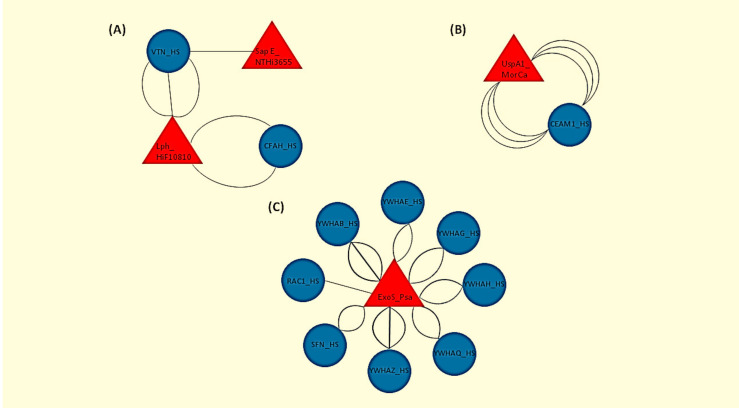
Graphical representation of the human-bacterial protein interactions as retrieved by the Host Pathogen Interaction Database version 3.0 (HPIDB 3.0). Human (Homo Sapiens, HS) proteins are shown in blue circles and bacterial strains are shown in red triangles. Each line represents a different experimental assay used to detect each interaction. (**A**) Hif strain 10810 interaction with CFAH; Hif strain 10810 and NTHi strain 3655 interaction with VTN. (**B**) MorCa interaction with CEAM1. (**C**) Psa interaction with YWHAB (tyrosine 3-monooxygenase/tryptophan 5-monooxygenase activation protein beta), YWHAE (tyrosine 3-monooxygenase/tryptophan 5-monooxygenase activation protein epsilon), YWHAG (tyrosine 3-monooxygenase/tryptophan 5-monooxygenase activation protein gamma), YWHAH (tyrosine 3-monooxygenase/tryptophan 5-monooxygenase activation protein eta), YWHAQ (tyrosine 3-monooxygenase/tryptophan 5-monooxygenase activation protein theta), YWHAZ (tyrosine 3-monooxygenase/tryptophan 5-monooxygenase activation protein zeta) and SFN (stratifin) (the last seven genes are all members of the 14-3-3 family of protein kinase C inhibitors), and RAC1 (ras-related C3 botulinum toxin substrate 1).

In total, 11 human proteins were found to interact with the bacterial pathogens as evidenced by the HPIDB 3.0 analysis. The genes expressing those 11 proteins were the following: CFAH (complement factor H), VTN (vitronectin), CEAM1 (carcinoembryonic antigen-related cell adhesion molecule 1), YWHAB (tyrosine 3-monooxygenase/tryptophan 5-monooxygenase activation protein beta), YWHAE (tyrosine 3-monooxygenase/tryptophan 5-monooxygenase activation protein epsilon), YWHAG (tyrosine 3-monooxygenase/tryptophan 5-monooxygenase activation protein gamma), YWHAH (tyrosine 3-monooxygenase/tryptophan 5-monooxygenase activation protein eta), YWHAQ (tyrosine 3-monooxygenase/tryptophan 5-monooxygenase activation protein theta), YWHAZ (tyrosine 3-monooxygenase/tryptophan 5-monooxygenase activation protein zeta) and SFN (stratifin) (the last seven genes are all members of the 14-3-3 family of protein kinase C inhibitors), and RAC1 (ras-related C3 botulinum toxin substrate 1). The exact type of host–pathogen interactions along with the experimental methods used in each case are shown in Table 1. A graphical representation of the host–pathogen interactomes is displayed in Figure 1.

### 3.2. ToppFun FEA Results for the CEACAM1, CFH, VTN, RAC1, YWHAB, YWHAE, YWHAG, YWHAH, YWHAQ, YWHAZ and SFN Genes, Relative to BP GOs, CC GOs and Diseases

In the context of BP GOs, the top five significantly enriched annotations pertained to protein insertion into mitochondrial membrane and mitochondrial membrane permeability, involved in apoptotic pathways. Interestingly, the genes related to these processes were found to be the seven genes of the 14-3-3 family in all five cases (Table 2). With respect to CC GOs, the top five annotations were related to membrane junctions and cell-substrate adhesion (Table 3). RAC1, CEACAM1 and all 14-3-3 family genes, except for SFN, were the predominant genes in these lists. Regarding diseases, the disorder where the input genes were over-represented was the lung adenocarcinoma, followed by the soft drusen and the bipolar and glomerular renal disorders (Table 4). Remarkably, five out of the seven genes that were found to relate to adenocarcinoma were also over-represented in the BP and CC annotations, with small differentiations in each case. This finding is highlighted in Table 5.

### 3.3. GeneCodis FEA Results for the CEACAM1, CFH, VTN, RAC1, YWHAB, YWHAE, YWHAG, YWHAH, YWHAQ, YWHAZ and SFN Genes Relative to BP GOs, CC GOs and KEGG Pathways

**Figure 2:**
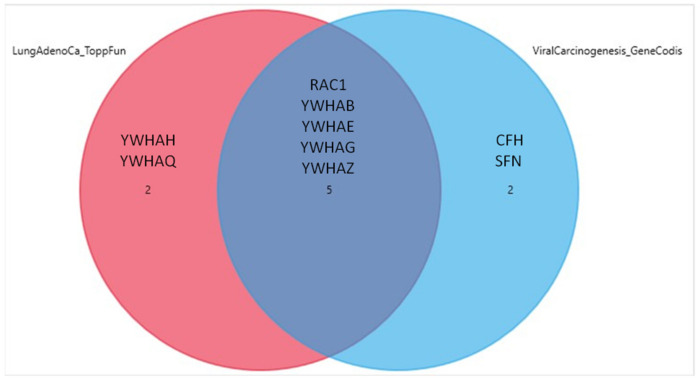
Venn diagram demonstrating that te gene overlaps between the lung adenocarcinoma and viral carcinogenesis ontologies, as retrieved from ToppFun and GeneCodis, respectively. Out of the seven genes over-represented in each annotation, five genes were common, as shown in the diagram.

In terms of BP GOs, the first two annotations were related to protein insertion into the mitochondrial membrane involved in apoptotic signaling pathway and membrane organization. The over-represented genes in this category belonged to the 14-3-3 family, as in the case of ToppFun (Table 6). The annotations with the higher level of confidence concerning cellular components were the extracellular exosomes and focal adhesion. All 11 genes were correlated to the exosomes annotation, while YWHAQ, YWHAZ, YWHAG, YWHAE, YWHAB and RAC1 were over-represented in the focal adhesion annotation (Table 7). Finally, the analysis with respect to the KEGG pathways revealed an involvement in the regulation of the cell cycle and a strong correlation with viral carcinogenesis. The genes that were found to correlate with the former are the seven genes of the 14-3-3 protein family, while the ones predicted to associate with the latter were the YWHAQ, YWHAZ, YWHAH, YWHAG, YWHAE, YWHAB and RAC1. The KEGG pathway predictions are shown in detail in Table 8. Of interest, five genes (RAC1, YWHAB, YWHAE, YWHAG and YWHAZ) were common in the lung adenocarcinoma and viral carcinogenesis ontologies, a finding which is presented in the form of a Venn diagram in Figure 2.

## 4. Discussion

### 4.1. Biological Interpretation of the Human-Gram (−) Pathogens Interactome

In total, 11 human proteins were identified as interactors with the three gram-negative pathogens. It has been reported that the lipoprotein binding factor H of *Hi* interacts with the human CFAH and VTN proteins, whereas the surface adhesion protein E is associated with VTN only. These types of interactions have been suggested to mediate the inhibition of the indirect complement activation pathway, serum resistance and pathogen adhesion to alveolar epithelium [14,22]. Human CEAM1 was found to interact with the UspA1 adhesin of *MorCa*. The ability of UspA1 to bind to CEAM1 by a trimeric coil has already been described [23]. UspA1 is an adhesive molecule, facilitating pathogen invasion in the respiratory epithelium and CEAM1 adhesive properties have also been reported [15,24]. Interaction of other gram-negative bacteria with CEAM1 has been found to mediate pathogen invasion; thus, this could also be the case for *MorCa* [24]. ExoS of *Psa* was associated with RAC1 and the members of the 14-3-3 protein family. ExoS has been shown to mediate RAC1 downregulation, thereby increasing *Psa* resistance to host cell defenses and blocking phagocytosis [16]. Furthermore, ExoS has been proven to bind all 14-3-3 family proteins, a procedure necessary for its activation [25,26]. This phenomenon is linked with increased *Psa* pathogenicity. Overall, these interactions could be interpreted as inducers of pathogen invasion and resistance thereby facilitating pathogenesis.

### 4.2. Gene Involvement in Apoptotic Pathways, Cellular Junctions, Cell Cycle, Carcinogenesis and Lung Adenocarcinoma: FEA Interpretation

The ToppFun FEA, relative to diseases, predicted a possible association of the RAC1, CFH and 5 of the 14-3-3 protein family genes, namely SFN, YWHAB, YWHAE, YWHAG and YWHAZ with lung adenocarcinoma. The GeneCodis results were in line with this prediction and further revealed an association of several of the interactome genes with the cell cycle. These findings suggest that the genes of interest could be linked to pathways which mediate the deregulation of cell cycle thus inducing carcinogenesis and, in this case, lung adenocarcinoma.

Additionally, ToppFun FEA relative to BP GOs revealed an association of all the 14-3-3 protein family genes to apoptotic pathways, whereas the same genes, together with RAC1 and CEACAM1 were over-represented in the CC GO annotations. Interestingly, FEA results from both databases shared substantial similarities. In the BP GOs and CC GOs, the common annotations were the “positive regulation of protein insertion into mitochondrial membrane involved in apoptotic signaling pathway” and “focal adhesion”, respectively. Of note, in each common annotation, the associated genes were the same, both in ToppFun and GeneCodis.

It has been reported that RAC1 contributes to the re-assembly and maintenance of the adherens junctions, thus preserving the epithelial integrity and the endothelial barrier function [27,28]. Moreover, it has been demonstrated that the inhibition of RAC1 in pulmonary endothelial cells leads to leaky junctions, a finding which highlights its protective role in the endothelial barrier [29]. Regarding the members of the 14-3-3 protein family (YWHAB, YWHAE, YWHAG, YWHAZ) and their role in cell junction regulation, previous studies have provided evidence that the binding of these proteins to protein kinase C or to Connexin 43 inhibits the activity of gap junctions [30,31]. It has also been suggested that the same proteins regulate cell adhesion, by interacting with integrin b1 [32]. When it comes to their involvement in apoptotic pathways, all 14-3-3 proteins have been found to bind the pro-apoptotic BCL family protein members BAD, BAX and BIN, inducing their inactivation and the inhibition of mitochondria mediated apoptosis [33,34,35,36]. Our findings, in line with these reported data, link the aforementioned proteins to the regulation of intercellular junctions, cell adhesion and apoptosis. The functional interplay of junctional proteins with cell adhesion and apoptosis and the contribution of this interaction to the pathogenesis and/or progression of various types of cancer have been well documented [37,38]. Moreover, *Psa* has been reported to contribute to endothelial and epithelial barrier disruption. One of the *Psa* proteins, LasB metalloprotease, induces interruption of intercellular and cell to matrix junctions of endothelial cells [39]. Additionally, *Psa* caused reorganization of tight junctions and reduction of transepithelial resistance of bronchial epithelial cells, in experiments conducted in vitro and in vivo [40]. These findings suggest that *Psa* could facilitate malignant behaviors such as transepithelial invasion and hematogenous metastasis. The results of our study suggest that human infection with the *Hi*, *MorCa* and *Psa* gram-negative bacteria could also be related with the pathogenesis and/or progression of lung adenocarcinoma possibly through the targeting of the epithelial cellular junctions and the subsequent deregulation of the cell adhesion and apoptotic pathways.

Regarding the enrichment of genes in the lung adenocarcinoma annotation, it has already been shown that RAC1 is important for lung cancer stem cell activity, and that its knockdown results in impaired proliferation, colony formation, adhesion, migration and invasion of human lung adenocarcinoma cells [41]. In tumor tissue from patients with lung adenocarcinoma and lung squamous cell carcinoma, RAC1 was over expressed, compared to normal tissue [42]. This finding was related to poor prognosis and high risk of lymphatic metastasis. In terms of viral carcinogenesis, RAC1 was found to facilitate HPV-8 related skin papilloma development [43]. On the other hand, the CFAH protein has been shown to increase the oncogenic action of adrenomedullin, a peptide that promotes tumor growth in various cancer types, including lung adenocarcinoma [44,45]. It has also been reported that complement factor H is over expressed in non-small cell lung cancer, blocking complement action on cancer cells [44]. As for SFN, it has been demonstrated that it is over expressed in invasive lung adenocarcinoma cell lines, compared to in situ adenocarcinoma or normal lung tissue and it induces proliferation of cancer cells [46]. Moreover, the 14-3-3 protein family members and, more specifically, the YWHAG and YWHAZ proteins have been linked to tumorigenesis and induction of malignant phenotypes, including cell growth, migration and invasion [47,48,49]. The same proteins are also involved in the regulation of cell cycle by interacting with the chk1 kinase on the one hand and by inhibiting the cdc25C protein on the other [50]. Infection with the gram-negative *E.Coli* has been associated with tumor growth, progression and metastasis of non-small cell lung cancer, by promoting lipid synthesis through the TLR4/9 pathway and, also, by facilitating cancer stem cell properties through the TLR4/IL-33 pathway [51,52,53,54]. Overall, published data have suggested a link of the proteins that occurred in our study to the cell cycle processes, carcinogenesis and malignant phenotypes.

### 4.3. Novelty, Weaknesses and Future Directions of Our Study

The novelty of our study lies in the unreported association of the *MorCa/Psa/Hi*-Human interactome with carcinogenesis and lung adenocarcinoma, as predicted by the in-silico analysis tools. In addition to this, we have identified specific BP GOs, CC GOs and KEGG pathways that are targeted by the human proteins interacting with the gram-negative pathogens. Our findings are also indicative of an overlap between the aforementioned annotations as the proteins that were found to associate with lung adenocarcinoma have also been related with the regulation of both the cell cycle and apoptotic pathways. Therefore, altered expression or function of these proteins due to the interaction with these pathogens could be followed by the disruption of the above processes, possibly leading to carcinogenesis. Additionally, these same proteins are also linked to cell junctions and adhesive components, which could be important for the adhesive, invasive and metastatic properties of a tumor. Altogether, this study does not only predict an association with lung adenocarcinoma but also suggests potential pathophysiological mechanisms that could lead to this clinical entity.

It should be mentioned that our in silico analysis has certain limitations since our results will have to be experimentally verified so as to test the aforementioned hypotheses. Our findings, however, provide the basis for an experimental rational to be followed in future clinical and translational studies with respect to the most common respiratory tract gram-negative pathogens in patients with lung adenocarcinoma and identification of the relationship of infection and carcinogenesis. It is worth mentioning that, in order to verify experimental data availability, we also performed a search in the gene expression omnibus (GEO) datasets, focusing on studies reporting on the association of the gram-negative bacteria with lung cancer or lung adenocarcinoma. Our search did not yield any transcriptomic data, thus future research is imperative in order to validate bioinformatic predictions and further expand our knowledge over the possible involvement of gram-negative bacteria respiratory infection with lung carcinogenesis.

## Figures and Tables

**Table 1:** The Human-MorCa/Psa/Hi interactome as retrieved by the Host Pathogen Interaction Database version 3.0 (HPIDB 3.0).

Human Protein	Pathogen	Bacterial Protein	Type of Interaction	Method
CEACAM1	MorCa	UspA1	direct interaction	X-ray scattering, Molecular sieving, enzyme-linked Immunosorbent Assay(ELISA), Far Western Blotting (FWB), isothermal titration, calorimetry, cosedimentation
CFAH	Hif 10810	Lipoprotein binding FH	direct interaction, physical association	FWB, ELISA
VTN	Hif 10810, NTHi strain 3655	Surface-adhesin protein E, Lipoprotein binding FH	direct interaction, physical association	ELISA, bio-layer interferometry, fluorescence activated cell sorting
YWHAG	Psa	Exoenzyme S	association	pull down, experimental interaction
YWHAE, YWHAQ, YWHAH	Psa	Exoenzyme S	association, direct interaction	pull down, experimental interaction
YWHAB, YWHAZ	Psa	Exoenzyme S	association, direct interaction	pull down, experimental interaction, X-ray crystallography
SFN	Psa	Exoenzyme S	association, direct interaction	pull down, experimental interaction
RAC1	Psa	Exoenzyme S	direct interaction	X-ray crystallography

**Table 2:** The top 5 annotations of the ToppFun FEA for BP GOs.

ID	BP	*P*	FDR B&H	FDR B&Y	Bonferroni	Genes from Input	Genes in Annotation
GO:1900740	positive regulation of protein insertion into mitochondrial membrane involved in apoptotic signaling pathway	1.094 × 10^−18^	6.744 × 10^−16^	5.189 × 10^−15^	1.349 × 10^−15^	YWHAB, YWHAE, YWHAG, YWHAH, YWHAQ, YWHAZ, SFN	27
GO:1900739	regulation of protein insertion into mitochondrial membrane involved in apoptotic signaling pathway	1.094 × 10^−18^	6.744 × 10^−16^	5.189 × 10^−15^	1.34910^−15^	YWHAB, YWHAE, YWHAG, YWHAH, YWHAQ, YWHAZ, SFN	27
GO:0001844	protein insertion into mitochondrial membrane involved in apoptotic signaling pathway	3.237 × 10^−18^	1.330 × 10^−15^	1.024 × 10^−14^	3.991 × 10^−15^	YWHAB, YWHAE, YWHAG, YWHAH, YWHAQ, YWHAZ, SFN	31
GO:1901030	positive regulation of mitochondrial outer membrane permeabilization involved in apoptotic signaling pathway	1.266 × 10^−17^	3.903 × 10^−15^	3.003 × 10^−14^	1.561 × 10^−14^	YWHAB, YWHAE, YWHAG, YWHAH, YWHAQ, YWHAZ, SFN	37
GO:1901028	regulation of mitochondrial outer membrane permeabilization involved in apoptotic signaling pathway	9.037 × 10^−17^	1.857 × 10^−14^	1.429 × 10^−13^	1.114 × 10^−13^	YWHAB, YWHAE, YWHAG, YWHAH, YWHAQ, YWHAZ, SFN	48

FDR: False Discovery Rate, B&H: Benjamini–Hochberg, B&Y: Benjamini–Yekutieli.

**Table 3:** The top 5 annotations of the ToppFun FEA for CC GOs.

ID	CC	*P*	FDR B&H	FDR B&Y	Bonferroni	Genes from Input	Genes in Annotation
GO:0005912	adherens junction	3.191 × 10^−9^	2.683 × 10^−7^	1.482 × 10^−6^	4.467 × 10^−7^	RAC1, CEACAM1, YWHAB, YWHAE, YWHAG, YWHAQ, YWHAZ	560
GO:0070161	anchoring junction	3.833 × 10^−9^	2.683 × 10^−7^	1.482 × 10^−6^	5.366 × 10^−7^	RAC1, CEACAM1, YWHAB, YWHAE, YWHAG, YWHAQ, YWHAZ	575
GO:0005925	focal adhesion	2.594 × 10^−8^	1.048 × 10^−6^	5.785 × 10^−6^	3.632 × 10^−6^	RAC1, YWHAB, YWHAE, YWHAG, YWHAQ, YWHAZ	411
GO:0030055	cell-substrate junction	2.993 × 10^−8^	1.048 × 10^−6^	5.785 × 10^−6^	4.190 × 10^−6^	RAC1, YWHAB, YWHAE, YWHAG, YWHAQ, YWHAZ	421
GO:0030054	cell junction	4.686 × 10^−8^	1.312 × 10^−6^	7.245 × 10^−6^	6.560 × 10^−6^	CEACAM1, RAC1, YWHAB, YWHAE, YWHAG, YWHAH, YWHAQ, YWHAZ	1352

FDR: False Discovery Rate, B&H: Benjamini–Hochberg, B&Y: Benjamini–Yekutieli.

**Table 4:** The top 5 annotations of the ToppFun FEA relative to diseases.

ID	Disease	*P*	FDR B&H	FDR B&Y	Bonferroni	Genes from Input	Genes in Annotation
C0152013	Adenocarcinoma of lung (disorder)	1.879 × 10^−6^	7.698 × 10^−4^	5.393 × 10^−3^	1.163 × 10^−3^	CFH, RAC1, SFN, YWHAB, YWHAE, YWHAG, YWHAZ	1123
C1720452	Soft drusen	2.487 × 10^−6^	7.698 × 10^−4^	5.393 × 10^−3^	1.540 × 10^−3^	CFH, VTN	4
C0268731	Renal glomerular disease	5.155 × 10^−5^	7.575 × 10^−3^	5.307 × 10^−2^	3.191 × 10^−2^	CFH, VTN, YWHAE	113
C0005586	Bipolar Disorder	6.287 × 10^−5^	7.575 × 10^−3^	5.307 × 10^−2^	3.892 × 10^−2^	RAC1, VTN, YWHAE, YWHAH, YWHAZ	723
C0017662	Glomerulonephritis, Membranoproliferative	6.310 × 10^−5^	7.575 × 10^−3^	5.307 × 10^−2^	3.906 × 10^−2^	CFH, VTN	18

FDR: False Discovery Rate, B&H: Benjamini–Hochberg, B&Y: Benjamini–Yekutieli.

**Table 5:** Combined results of the ToppFun FEA: Overlaps of the annotated genes between the Lung adenocarcinoma, BP GOs and CC GOs.

Lung Adenocarcinoma	Apoptotic Processes	Focal Adhesion	Adherens Junctions	Anchoring Junctions	Cell-Substrate Junctions	Cell Junction
*RAC1*	−	+	+	+	+	+
*CFH*	−	−	−	−	−	−
*SFN*	+	−	−	−	−	−
*YWHAB*	+	+	+	+	+	+
*YWHAE*	+	+	+	+	+	+
*YWHAG*	+	+	+	+	+	+
*YWHAZ*	+	+	+	+	+	+

**Table 6:** The top 5 annotations identified by the GeneCodis FEA for BP GOs.

Terms	Annotations	Term’s Genes Found	Term’s Genes	Genes Universe	Hyp pVal	Hyp pValAdj	Genes
positive regulation of protein insertion into mitochondrial membrane involved in apoptotic signaling pathway	GO:1900740	7	27	17898	2.50 × 10^−18^	5.01 × 10^−16^	YWHAQ, YWHAZ, YWHAH, YWHAG, YWHAE, YWHAB, SFN
membrane organization	GO:0061024	7	135	17898	3.82 × 10^−13^	3.82 × 10^−11^	YWHAQ, YWHAZ, YWHAH, YWHAG, YWHAE, YWHAB, SFN
protein targeting	GO:0006605	5	48	17898	5.11 × 10^−11^	3.41 × 10^−9^	YWHAQ, YWHAZ, YWHAG, YWHAE, YWHAB
substantia nigra development	GO:0021762	3	46	17898	2.58 × 10^−6^	0.000129202	YWHAQ, YWHAH, YWHAE
negative regulation of protein dephosphorylation	GO:0035308	2	10	17898	1.54 × 10^−5^	0.000513729	YWHAE, YWHAB

Hyp pVal: Hypergeometric *p* values; Hyp pVal Adj: Hypergeometric *p* values adjusted by the Benjamini and Hochberg FDR.

**Table 7:** The top 5 annotations identified by the GeneCodis FEA for CC GOs.

Terms	Annotations	Term’s Genes Found	Term’s Genes	Genes Universe	Hyp pVal	Hyp pValAdj	Genes
extracellular exosome	GO:0070062	11	2169	18876	4.51 × 10^−11^	2.98 × 10^−9^	YWHAQ, YWHAZ, YWHAH, YWHAG, YWHAE, YWHAB, VTN, CEACAM1, RAC1, CFH, SFN
focal adhesion	GO:0005925	6	410	18876	4.27 × 10^−8^	1.41 × 10^−6^	YWHAQ, YWHAZ, YWHAG, YWHAE, YWHAB, RAC1
melanosome	GO:0042470	4	100	18876	2.38 × 10^−7^	5.23 × 10^−6^	YWHAZ, YWHAE, YWHAB, RAC1
mitochondrion	GO:0005739	7	1538	18876	5.79 × 10^−6^	9.55 × 10^−5^	YWHAQ, YWHAZ, YWHAH, YWHAG, YWHAE, YWHAB, SFN
blood microparticle	GO:0072562	3	143	18876	6.72 × 10^−5^	0.00088691	YWHAZ, VTN, CFH

Hyp pVal: Hypergeometric *p* values; Hyp pVal Adj: Hypergeometric *p* values adjusted by the Benjamini and Hochberg FDR.

**Table 8:** The top 5 annotations identified by the GeneCodis FEA for KEGG pathways.

Terms	Annotations	Term’s Genes Found	Term’s Genes	Genes Universe	Hyp pVal	Hyp pValAdj	Genes
Cell cycle	hsa04110	7	124	8013	2.07 × 10−11	1.20 × 10−9	YWHAQ, YWHAZ, YWHAH, YWHAG, YWHAE, YWHAB, SFN
Viral carcinogenesis	hsa05203	7	201	8013	6.34 × 10−10	1.23 × 10−8	YWHAQ, YWHAZ, YWHAH, YWHAG, YWHAE, YWHAB, RAC1
PI3K-Akt signaling pathway	hsa04151	8	354	8013	5.59 × 10−10	1.62 × 10−8	YWHAQ, YWHAZ, YWHAH, YWHAG, YWHAE, YWHAB, VTN, RAC1
Oocyte meiosis	hsa04114	6	128	8013	2.94 × 10−9	4.27 × 10−8	YWHAQ, YWHAZ, YWHAH, YWHAG, YWHAE, YWHAB
Hepatitis C	hsa05160	6	155	8013	9.37 × 10−9	9.06 × 10−8	YWHAQ, YWHAZ, YWHAH, YWHAG, YWHAE, YWHAB

Hyp pVal: Hypergeometric *p* values; Hyp pVal Adj: Hypergeometric *p* values adjusted by the Benjamini and Hochberg FDR.

## Data Availability

Data is contained within the article.

## References

[1] Sriram K.B., Cox A.J., Clancy R.L., Slack M.P.E., Cripps A.W. (2018). Nontypeable Haemophilus influenzae and chronic obstructive pulmonary disease: A review for clinicians. Crit. Rev. Microbiol..

[2] Vickery T.W., Ramakrishnan V.R. (2017). Bacterial Pathogens and the Microbiome. Otolaryngol. Clin. N. Am..

[3] Atkinson H., Wallis S., Coatesworth A.P. (2015). Acute otitis media. Postgrad. Med..

[4] Fujitani S., Sun H.-Y., Yu V.L., Weingarten J.A. (2011). Pneumonia due to Pseudomonas aeruginosa: Part I: Epidemiology, Clinical Diagnosis, and Source. Chest.

[5] Riesbeck K. (2020). Complement evasion by the human respiratory tract pathogens *Haemophilus influenzae* and *Moraxella catarrhalis*. FEBS Lett..

[6] Whittaker R., Economopoulou A., Dias J.G., Bancroft E., Ramliden M., Celentano L.P., Steindl G., Martiny D., Grammens T., Georgieva T. (2017). Epidemiology of invasive Haemophilus influenzae disease, Europe, 2007–2014. Emerg. Infect. Dis..

[7] Soeters H.M., Blain A., Pondo T., Doman B., Farley M.M., Harrison L.H., Lynfield R., Miller L., Petit S., Reingold A. (2018). Current Epidemiology and Trends in Invasive Haemophilus influenzae Disease—United States, 2009–2015. Clin. Infect. Dis..

[8] Blakeway L.V., Tan A., Peak I.R., Seib K.L. (2017). Virulence determinants of Moraxella catarrhalis: Distribution and considerations for vaccine development. Microbiology.

[9] Murphy T.F., Brauer A.L., Grant B.J.B., Sethi S. (2005). Moraxella catarrhalis in chronic obstructive pulmonary disease: Burden of disease and immune response. Am. J. Respir. Crit. Care Med..

[10] Tan T.T., Mörgelin M., Forsgren A., Riesbeck K. (2007). *Haemophilus influenzae* Survival during Complement-Mediated Attacks Is Promoted by *Moraxella catarrhalis* Outer Membrane Vesicles. J. Infect. Dis..

[11] Rodrigo-Troyano A., Sibila O. (2017). The respiratory threat posed by multidrug resistant Gram-negative bacteria. Respirology.

[12] Wilson R., Aksamit T., Aliberti S., De Soyza A., Elborn J., Goeminne P., Hill A.T., Menendez R., Polverino E. (2016). Challenges in managing Pseudomonas aeruginosa in non-cystic fibrosis bronchiectasis. Respir. Med..

[13] Hallström T., Blom A.M., Zipfel P.F., Riesbeck K. (2009). Nontypeable *Haemophilus influenzae* Protein E Binds Vitronectin and Is Important for Serum Resistance. J. Immunol..

[14] Fleury C., Su Y.-C., Hallström T., Sandblad L., Zipfel P.F., Riesbeck K. (2014). Identification of a *Haemophilus influenzae* Factor H–Binding Lipoprotein Involved in Serum Resistance. J. Immunol..

[15] Spaniol V., Heiniger N., Troller R., Aebi C. (2008). Outer membrane protein UspA1 and lipooligosaccharide are involved in invasion of human epithelial cells by Moraxella catarrhalis. Microbes Infect..

[16] Würtele M., Wolf E., Pederson K.J., Buchwald G., Ahmadian M.R., Barbieri J.T., Wittinghofer A. (2001). How the Pseudomonas aeruginosa ExoS toxin downregulates Rac. Nat. Struct. Mol. Biol..

[17] Paulsson M., Riesbeck K. (2018). How bacteria hack the matrix and dodge the bullets of immunity. Eur. Respir. Rev..

[18] Ammari M.G., Gresham C.R., McCarthy F.M., Nanduri B. (2016). HPIDB 2.0: A curated database for host-pathogen interactions. Database.

[19] Bateman A. (2019). UniProt: A worldwide hub of protein knowledge. Nucleic Acids Res..

[20] Chen J., Bardes E.E., Aronow B.J., Jegga A.G. (2009). ToppGene Suite for gene list enrichment analysis and candidate gene prioritization. Nucleic Acids Res..

[21] Tabas-Madrid D., Nogales-Cadenas R., Pascual-Montano A. (2012). GeneCodis3: A non-redundant and modular enrichment analysis tool for functional genomics. Nucleic Acids Res..

[22] Al-Jubair T., Mukherjee O., Oosterhuis S., Singh B., Su Y.-C., Fleury C., Blom A.M., Törnroth-Horsefield S., Riesbeck K. (2015). Haemophilus influenzae Type f Hijacks Vitronectin Using Protein H To Resist Host Innate Immunity and Adhere to Pulmonary Epithelial Cells. J. Immunol..

[23] Conners R., Hill D.J., Borodina E., Agnew C., Daniell S.J., Burton N.M., Sessions R.B., Clarke A.R., Catto L., Lammie D. (2008). The Moraxella adhesin UspA1 binds to its human CEACAM1 receptor by a deformable trimeric coiled-coil. EMBO J..

[24] Hill D.J., Edwards A.M., Rowe H.A., Virji M. (2005). Carcinoembryonic antigen-related cell adhesion molecule (CEACAM)-binding recombinant polypeptide confers protection against infection by respiratory and urogenital pathogens. Mol. Microbiol..

[25] Henriksson M.L., Trollér U., Hallberg B. (2000). 14-3-3 proteins are required for the inhibition of Ras by exoenzyme S. Biochem. J..

[26] Henriksson M.L., Francis M.S., Peden A., Aili M., Stefansson K., Palmer R., Aitken A., Hallberg B. (2002). A nonphosphorylated 14-3-3 binding motif on exoenzyme S that is functional in vivo. Eur. J. Biochem..

[27] Timmerman I., Heemskerk N., Kroon J., Schaefer A., Van Rijssel J., Hoogenboezem M., Van Unen J., Goedhart J., Gadella T.W.J., Yin T. (2015). A local VE-cadherin and Trio-based signaling complex stabilizes endothelial junctions through Rac1. J. Cell Sci..

[28] Ehrlich J.S., Hansen M.D., Nelson W.J. (2002). Spatio-Temporal Regulation of Rac1 Localization and Lamellipodia Dynamics during Epithelial Cell-Cell Adhesion. Dev. Cell.

[29] Wojciak-Stothard B., Torondel B., Zhao L., Renné T., Leiper J.M. (2009). Modulation of Rac1 Activity by ADMA/DDAH Regulates Pulmonary Endothelial Barrier Function. Mol. Biol. Cell..

[30] Smyth J.W., Zhang S.-S., Sanchez J.M., Lamouille S., Vogan J.M., Hesketh G.G., Hong T., Tomaselli G.F., Shaw R.M. (2014). A 14-3-3 Mode-1 Binding Motif Initiates Gap Junction Internalization During Acute Cardiac Ischemia. Traffic.

[31] Nguyen T.A., Takemoto L.J., Takemoto D.J. (2004). Inhibition of gap junction activity through the release of the C1B domain of protein kinase Cgamma (PKCgamma) from 14-3-3: Identification of PKCgamma-binding sites. J. Biol. Chem..

[32] Han D.C., Rodriguez L.G., Guan J.L. (2000). Identification of a novel interaction between integrin β1 and 14-3-3β. Oncogene.

[33] Zha J., Harada H., Yang E., Jockel J., Korsmeyer S.J. (1996). Serine Phosphorylation of Death Agonist BAD in Response to Survival Factor Results in Binding to 14-3-3 Not BCL-XL. Cell.

[34] Masters S.C., Yang H., Datta S.R., Greenberg M.E., Fu H. (2001). 14-3-3 Inhibits Bad-Induced Cell Death through Interaction with Serine-136. Mol. Pharmacol..

[35] Porter G., Khuri F.R., Fu H. (2006). Dynamic 14-3-3/client protein interactions integrate survival and apoptotic pathways. Semin. Cancer Biol..

[36] Gardino A.K., Yaffe M.B. (2011). 14-3-3 proteins as signaling integration points for cell cycle control and apoptosis. Semin. Cell Dev. Biol..

[37] Knights A.J., Funnell A.P.W., Crossley M., Pearson R.C. (2012). Holding Tight: Cell Junctions and Cancer Spread. Trends Cancer Res..

[38] Bhat A.A., Uppada S., Achkar I.W., Hashem S., Yadav S.K., Shanmugakonar M., Al-Naemi H.A., Haris M., Uddin S. (2019). Tight Junction Proteins and Signaling Pathways in Cancer and Inflammation: A Functional Crosstalk. Front. Physiol..

[39] Beaufort N., Corvazier E., Mlanaoindrou S., De Bentzmann S., Pidard D. (2013). Disruption of the Endothelial Barrier by Proteases from the Bacterial Pathogen Pseudomonas aeruginosa: Implication of Matrilysis and Receptor Cleavage. PLoS ONE.

[40] Rejman J., Di Gioia S., Bragonzi A., Conese M. (2007). Pseudomonas aeruginosaInfection Destroys the Barrier Function of Lung Epithelium and Enhances Polyplex-Mediated Transfection. Hum. Gene Ther..

[41] Akunuru S., Palumbo J., Zhai Q.J., Zheng Y. (2011). Rac1 Targeting Suppresses Human Non-Small Cell Lung Adenocarcinoma Cancer Stem Cell Activity. PLoS ONE.

[42] Liu Y., Wang Y., Zhang Y., Miao Y., Zhao Y., Zhang P.-X., Jiang G., Zhang J.-Y., Han Y., Lin X.-Y. (2009). Abnormal expression of p120-catenin, E-cadherin, and small GTPases is significantly associated with malignant phenotype of human lung cancer. Lung Cancer.

[43] Deshmukh J., Pofahl R., Pfister H., Haase I. (2016). Deletion of epidermal Rac1 inhibits HPV-8 induced skin papilloma formation and facilitates HPV-8- and UV-light induced skin carcinogenesis. Oncotarget.

[44] Ajona D., Castaño Z., Garayoa M., Zudaire E., Pajares M.J., Martínez A., Cuttitta F., Montuenga L.M., Pío R. (2004). Expression of Complement Factor H by Lung Cancer Cells. Cancer Res..

[45] Miller M.J., Martínez A., Unsworth E.J., Thiele C.J., Moody T.W., Elsasser T., Cuttitta F. (1996). Adrenomedullin expression in human tumor cell lines. Its potential role as an autocrine growth factor. J. Biol. Chem..

[46] Shiba-Ishii A., Kano J., Morishita Y., Sato Y., Minami Y., Noguchi M. (2011). High expression of stratifin is a universal abnormality during the course of malignant progression of early-stage lung adenocarcinoma. Int. J. Cancer.

[47] Raungrut P., Wongkotsila A., Champoochana N., Lirdprapamongkol K., Svasti J., Thongsuksai P. (2018). Knockdown of 14-3-3γ Suppresses Epithelial–Mesenchymal Transition and Reduces Metastatic Potential of Human Non-small Cell Lung Cancer Cells. Anticancer. Res..

[48] Khorrami A., Bagheri M.S., Tavallaei M., Gharechahi J. (2017). The functional significance of 14-3-3 proteins in cancer: Focus on lung cancer. Horm. Mol. Biol. Clin. Investig..

[49] Tong S., Xia T., Fan K., Jiang K., Zhai W., Li J.-S., Wang S.-H., Wang J.-J. (2016). Loss of Par3 promotes lung adenocarcinoma metastasis through 14-3-3ζ protein. Oncotarget.

[50] Dalal S.N., Yaffe M.B., DeCaprio J.A. (2004). 14-3-3 Family Members Act Coordinately to Regulate Mitotic Progression. Cell Cycle.

[51] Gowing S.D., Chow S.C., Cools-Lartigue J.J., Chen C.B., Najmeh S., Goodwin-Wilson M., Jiang H.Y., Bourdeau F., Beauchamp A., Angers I. (2019). Gram-Negative Pneumonia Augments Non–Small Cell Lung Cancer Metastasis through Host Toll-like Receptor 4 Activation. J. Thorac. Oncol..

[52] Chow S.C., Gowing S.D., Cools-Lartigue J.J., Chen C.B., Berube J., Yoon H.W., Chan C.H.F., Rousseau M.C., Bourdeau F., Giannias B. (2015). Gram negative bacteria increase non-small cell lung cancer metastasis via toll-like receptor 4 activation and mitogen-activated protein kinase phosphorylation. Int. J. Cancer..

[53] Sun M., Bai Y., Zhao S., Liu X., Gao Y., Wang L., Liu B., Ma D., Ma C. (2018). Gram-negative bacteria facilitate tumor progression through TLR4/IL-33 pathway in patients with non-small-cell lung cancer. Oncotarget.

[54] Ye M., Gu X., Han Y., Jin M., Ren T. (2016). Gram-negative bacteria facilitate tumor outgrowth and metastasis by promoting lipid synthesis in lung cancer patients. J. Thorac. Dis..

